# Correlation Between HbA1c Levels and Mortality Rates in Hospitalized COVID-19 Patients

**DOI:** 10.7759/cureus.31347

**Published:** 2022-11-10

**Authors:** Ankur Bhagat, Luke Buxton, Mufadda Hasan

**Affiliations:** 1 Internal Medicine, Arrowhead Regional Medical Center, Colton, USA; 2 Pulmonary and Critical Care Medicine, Arrowhead Regional Medical Center, Colton, USA

**Keywords:** correlation between a1c and mortality, inpatient covid outcomes, a1c, diabetes, covid-19, covid-19 and mortality

## Abstract

Objective

Throughout the coronavirus disease 2019 (COVID-19) pandemic, multiple factors have been associated with poor prognosis for those infected by the severe acute respiratory syndrome coronavirus 2 (SARS-CoV-2) virus. Age, obesity, and medical comorbidities have been linked to poor outcomes, including admission to the intensive care unit (ICU), acute renal failure, stroke, myocardial infarction (MI), mechanical ventilation, and even death for hospitalized COVID-19 patients. Although diabetes mellitus (DM) has also been included in this set of medical comorbidities, there have been inconsistencies in the currently available body of literature, suggesting that mortality rates may or may not be correlated to elevated glycosylated hemoglobin (HbA1c) levels. This study aims to determine whether there is a correlation or trend between a range of HbA1c values and in-hospital mortality among patients admitted to the hospital with a COVID-19 diagnosis.

Materials and methods

This study was a retrospective review of electronic medical records at Arrowhead Regional Medical Center in Colton, CA. Any patient above the age of 18 admitted to the hospital during a predetermined time frame, with either a positive COVID-19 PCR test on admission or during their hospital stay, was included in the study. These medical charts were reviewed for HbA1c values during admission or within three months prior to admission. In-hospital mortality was then recorded for each medical record with an available HbA1c value. Hospital discharge summaries were used to delineate comorbidities, including chronic kidney disease, cerebrovascular disease, coronary artery disease, congestive heart failure, cancer history, or history of deep vein thrombosis/pulmonary embolism among the patients included in the study. Average HbA1c values were recorded for the mortality and non-mortality groups, and their statistical significance was calculated.

Results

In this retrospective study, HbA1c levels were compared to mortality rates among adult patients admitted to the hospital with a concurrent COVID-19 diagnosis. From the analysis conducted, those with higher HbA1c levels did not have an increased rate of in-hospital mortality, and those with lower HbA1c levels did not have a decreased rate of in-hospital mortality. Comorbidity data as a confounding factor was also reviewed and excluded from the final analysis. The SARS-CoV-2 vaccine was also excluded as a confounder in this study by selecting a specific time frame for data collection. Based on our results, we propose that HbA1c levels likely have little to minimal correlation with mortality rates among hospitalized COVID-19 patients.

Conclusion

In this study, we show that HbA1c levels, regardless of concurrent comorbidities, are unlikely to be correlated to mortality rates among hospitalized COVID-19 patients. HbA1c levels should not be used as a marker for whether these patients should be admitted to the hospital for further inpatient management or discharged from the emergency department.

## Introduction

SARS-CoV-2, a single-stranded RNA virus in the coronavirus family that causes the COVID-19 infection, was first discovered in Wuhan, China, on January 7, 2020 [1,2]. Coronavirus 2019 (COVID-19) began to spread rapidly across borders through person-to-person transmission, and the first case in the US was reported on January 20, 2020, in Washington State [2]. By March 11, 2020, the World Health Organization had declared the outbreak, caused by the SARS-CoV-2 virus, a global pandemic [3]. Toward the end of 2020, there were over 80 million documented infections and 1.8 million deaths globally that were attributed to COVID-19 [1]. Those infected by the virus displayed various clinical signs, symptoms, and hospital courses, including mild respiratory illnesses, asymptomatic infections, and even severe pneumonia with multiorgan failure leading to death [4-6]. With this widespread disease activity and variation in disease severity, it became imperative to uncover associated factors that might lead to worse clinical outcomes among individuals. After a significant analysis of COVID-19 trends from hospitals globally, a subset of factors that increased the risk of acquiring severe SARS-CoV-2 infection was outlined. Older age and male gender, in addition to co-morbid conditions, such as cardiovascular disease, obesity, hypertension, and diabetes mellitus, have since been associated with worse outcomes in patients with COVID-19 [7-9]. These aforementioned co-morbidities have been denoted as risk factors for elevated morbidity and mortality in COVID-19 patients [1,10]. Diabetes mellitus has been specifically labeled as an independent risk factor for worse clinical outcomes among those admitted with SARS-CoV-2 infection. Patients with diabetes required hemodialysis, extensive antibiotic therapy, and mechanical ventilation at higher rates; additionally, these patients had an increased length of hospital stay [1,11]. Although diabetes has been shown to have worse outcomes in hospitalized COVID-19 patients, there has been some debate on whether poor long-term glycemic control and subsequently elevated glycosylated hemoglobin (HbA1c) levels (which signifies the average blood glucose levels over the past three months) correlate strongly with increased mortality [5,12-14]. A study conducted by Wu, et al. in Wuhan, China, claimed that elevated glucose levels at the time of admission were independently associated with an increased risk of progression to critical illness and death, including in-hospital mortality [15]. Aggressive glucose control was associated with shorter lengths of stay and overall decreased mortality rates as compared with poor glucose control [1]. On the other hand, a study conducted by Mehta et al. in 2021 showed that outpatient glucose control, inpatient glucose control, average glucose during hospital admission, or even level of HbA1c did not correlate with ventilator requirement, ICU admission, or mortality in patients hospitalized with COVID-19 [16]. Thus, there have been variations in data reported regarding diabetes, its specific markers, and its association or lack thereof with the severity of COVID-19 infection.

HbA1c has been shown to objectively delineate diabetes severity and overall glycemic control over an average of three months [14]. HbA1c levels greater than or equal to 9% have been associated with a significantly increased risk of hospitalization in COVID-19 patients [17]. Naturally, with a higher risk of hospitalization and clinically adverse events, one may assume that mortality would also be increased in these patients. A multicenter review performed by Kristan et al. demonstrates increased mortality rates in those with elevated HbA1c levels [1]. However, data presented in a study by Randhawa et al. in 2021, and subsequently by Patel, et al. in a single-center retrospective study, showed that there may not be any correlation between elevated HbA1c levels and mortality in hospitalized patients with a COVID-19 diagnosis; although elevated HbA1c levels have been linked to worse clinical outcomes and complications, mortality was not one of them [5,18]. Given the conflicting results of prior studies, more research is needed to illuminate the true risks that poorly controlled diabetes has on SARS-CoV-2 infection severity and in-hospital mortality. This may have a profound effect on whether or not a symptomatic patient who presents to the emergency department qualifies for admission to the hospital. Thus, our single-centered, retrospective study attempts to evaluate this topic further.

## Materials and methods

Study design

The goal of this study was to find the correlation between adult patients admitted to either medical floors or ICU carrying a diagnosis of COVID-19 (confirmed by polymerase chain reaction (PCR) testing of nasopharyngeal swabs for SARS-CoV-2 virus) with their documented HbA1c level. This study was a retrospective review of electronic medical records at Arrowhead Regional Medical Center in Colton, CA. It was reviewed and approved by the Arrowhead Regional Medical Center Institutional Review Board (Protocol# 22-35) on June 17, 2022. All medical records and reviews complied with HIPAA regulations. Patient identifiers were strictly limited to the medical record number, age, sex, ethnicity, body mass index (BMI), past medical history, comorbidities, and outcome of admission (disposition).

Inclusion and exclusion criteria

All the electronic charts were first filtered with basic search criteria. Any patient that was above the age of 18 and was admitted to the hospital (Floors/Wards or ICU) from March 1, 2020, to December 1, 2020, with either a positive COVID-19 PCR test on admission or during their hospital stay was included in the initial search. This was achieved through searching International Classification of Diseases (ICD) codes relevant to a COVID-19 or acute hypoxic respiratory failure due to COVID-19 diagnosis. The reason for choosing this specific time frame was to exclude the effect of the SARS-CoV-2 vaccine as a confounder to mortality data since the vaccine was first available for distribution in December 2020 [19]. Exclusion criteria for selection were any patient who was not admitted to the hospital, was admitted outside of the time frame listed above, was under the age of 18, or did not carry the diagnosis of COVID-19 infection during that specific hospital stay. 

Data collection

Each of the selected charts was meticulously reviewed for an HbA1c level obtained during or within three months of the patient’s hospital stay. In-hospital mortality was then recorded for each medical record with an available HbA1c value, regardless of how high or low that value was. Hospital discharge summaries were used to aid in the collection of that individual’s medical history to delineate comorbidities, including chronic kidney disease, cerebrovascular disease, coronary artery disease, congestive heart failure, cancer history, or history of deep vein thrombosis/pulmonary embolism. Average HbA1c values were recorded for the mortality and non-mortality groups and their statistical significance was calculated using a p-value. After data were collected, HbA1c values were stratified in different groups and comorbidity data was excluded from the final calculation of mean values. The data were subsequently organized into a tabular format.

## Results

Data from electronic medical records at our institution were reviewed from March 1, 2020, to December 1, 2020. Those cases that were above the age of 18, were admitted to the hospital on the floors or ICU, and had a diagnosis of COVID-19 were examined. There were 487 records reviewed in total. There were 193 females (40%) and 294 males (60%) in this cohort, and the median age was 58. Of those 487 cases, 379 of them were included in this study as they had a valid HbA1c level drawn during their hospital stay or within three months prior to their hospital stay. Out of those 379 cases, 87 (22.9%) suffered in-hospital mortality. The average age of these patients was 62.5. The average HbA1c level for mortality cases was 7.66%. HbA1c values for all the cases included were divided numerically from <5% to >14%. The groupings were in intervals of 1 percentage point. For example, 5.0-5.9% was one interval group while 6.0-6.9% was the next. The lowest mortality rate was 15.8% in the 11.0-11.9% HbA1c group. There were 19 cases documented in this group and three deaths. The highest mortality rate was in the > 14% HbA1c group at 33.3%; however, there were only three documented cases and one death. All the other groupings had a mortality rate between 21%-27%. The mortality rates in the 5.0-5.9%, 6.0-6.9%, and 7.0-7.9% HbA1c groups were 21.7%, 22%, and 25.5% respectively. The mortality rates in the 8.0-8.9%, 9.0-9.9%, and 10.0-10.9% HbA1c groups were 25.9%, 20.8%, and 26.9% respectively (Table 1).

**Table 1:** Correlation between stratified HbA1c values and hospital mortality among COVID-19 patients HbA1c = glycosylated hemoglobin

Totals including patients with any comorbidities	Total #	Deaths
HbA1c values	379	87 (Total)
< 5%	8	2 (25%)
5-5.9%	92	20 (21.7%)
6-6.9%	109	24 (22%)
7-7.9%	47	12 (25.5%)
8-8.9%	27	7 (25.9%)
9-9.9%	24	5 (20.8%)
10-10.9%	26	7 (26.9%)
11-11.9%	19	3 (15.8%)
12-12.9%	12	3 (25%)
13-13.9%	12	3 (25%)
> 14%	3	1 (33.3%)

Of the 87 in-hospital deaths that occurred in this cohort, 26 (29.9%) patients had at least one documented comorbidity. The remaining 61 (70.1%) patients did not have any documented comorbidity. The comorbidities that were looked for in each case included a history of cerebrovascular disease, coronary artery disease, congestive heart failure, chronic kidney disease, cancer, or deep vein thrombosis (DVT)/ pulmonary embolism (PE). Of those that died in the 5.0-5.9%, 6.0-6.9%, 7.0-7.9%, 8.0-8.9% HbA1c groups, 35%, 33.3%, 25%, 28.6% respectively had at least 1 comorbidity. To account for comorbidities as a confounder in this study, data were filtered to exclude patients with any of the above-mentioned comorbidities. There was a total of 301 cases in that 387 original cohort used in the study for which there was no comorbidity documented as reviewed by physician discharge summaries and progress notes. From this group, there were 61 (20.3%) total deaths. Groupings for each interval were similar to the ones above. The lowest mortality rate was 15.4% in the 9.0-9.9% HbA1c group. There were 13 cases documented in this group and two deaths. The highest mortality rate was in the >14% HbA1c group at 33.3%; however, as mentioned earlier, there were only three documented cases and one death. The <5% HbA1c group and the 11.0-11.9% HbA1c group both had a mortality rate of 16.7%. The mortality rates in the 5.0-5.9%, 6.0-6.9%, 7.0-7.9%, and 8.0-8.9% HbA1c groups were 18.3%, 18%, 25.8%, and 25% respectively. Additionally, the mortality rates in the 10.0-10.9%, 11.0-11.9%, 12.0-12.9%, and 13.0-13.9% HbA1c groups were 25%, 16.7%, 20%, and 25% respectively (Table 2). In the end, when looking at the entire data collectively, the average HbA1c for the mortality group was 7.66 + 2.36, whereas the HbA1c for the non-mortality group was 7.65 + 2.34. The p-value was 0.964, indicating that there was no statistical significance between the HbA1c values of those that died in the hospital with a COVID-19 diagnosis versus those that did not.

**Table 2:** Correlation between stratified HbA1c levels and hospital mortality among COVID-19 patients after excluding cases with a prior history of medical comorbidities HbA1c = glycosylated hemoglobin

Totals without including patients with any comorbidities	Total #	Deaths
HbA1c values	301	61 (Total)
< 5%	6	1 (16.7%)
5-5.9%	71	13 (18.3%)
6-6.9%	89	16 (18%)
7-7.9%	31	8 (25.8%)
8-8.9%	24	6 (25%)
9-9.9%	13	2 (15.4%)
10-10.9%	24	6 (25%)
11-11.9%	18	3 (16.7%)
12-12.9%	10	2 (20%)
13-13.9%	12	3 (25%)
> 14%	3	1 (33.3%)

## Discussion

Our focus in this single-center, retrospective, observational study was to understand the correlation between HbA1c levels and mortality among patients admitted to the hospital with a diagnosis of COVID-19. HbA1c levels generally show the trend of glycemic control for the past three months, therefore illuminating the severity of an individual’s diabetes [14]. Since diabetes is a significant risk factor for a complicated hospital course and the severity of SARS-CoV-2 infection, we aimed to determine whether poorly controlled diabetes leads to increased rates of mortality [1,11]. Unraveling this data can potentially help individuals stratify their own risk of acquiring a severe disease and encourage them to take extra precautions to mitigate their risk of contracting COVID-19. Additionally, it could provide clinicians insight into which patients may benefit from admission to the hospital and whether they should be admitted to an ICU setting or general medical floor. Of note, prior studies have claimed that elevated HbA1c correlates with increased mortality; however, other studies do not suggest a correlation between HbA1c levels with mortality [1,5]. Our study was designed to evaluate this topic further and provide clarity by presenting data specific to our population at a county hospital in San Bernardino, California. The specific time interval chosen, which may be different from prior studies, was chosen to limit the confounding effects of the SARS-CoV-2 vaccination, which became available in December 2020 [19]. Thus, the study time frame ended on December 1, 2020. Based on the data obtained, there does not appear to be a clear correlation between HbA1c values and in-hospital mortality, regardless of whether a patient was admitted to the ICU or general medical floor. For each subset of the HbA1c level, the mortality rates were relatively comparable. This was further evidenced by the statistical insignificance of the average HbA1c values between the mortality group and the non-mortality group. The average HbA1c for the mortality group was 7.66 + 2.36, whereas the HbA1c for the non-mortality group was 7.65 + 2.34, with a p-value of 0.964. Notably, the lowest mortality rate was seen in the 11.0-11.9% HbA1c group, which generally signifies very poorly controlled diabetes. Even after accounting for comorbidities, which may have been a confounder, the mortality rates were not substantially different among each HbA1c subset. The mortality rate was 22.9% overall and when patients with a history of any medical comorbidities were excluded, the mortality rate dropped only slightly to 20.3%. The average HbA1c level among the mortality cases was 7.66%, which indicated moderately poor glycemic control. However, given the range of HbA1c levels seen in this specific population (ranging as high as 14%), it is difficult to say that higher HbA1c levels were associated with greater mortality. In fact, there may not be a significant correlation at all.

One specific point to consider is that this study was largely inpatient-focused. It did not include outpatient management and control of diabetes prior to hospital admission. Patients with a higher HbA1c may likely have been on diabetic medications previously, including insulin. It is unclear how being on diabetic medications, such as insulin and metformin, in the outpatient setting would affect these results. As noted by Patel et al. in their study, HbA1c values could potentially be skewed in certain populations such as those with chronic kidney disease and/or in patients with anemia, which may affect the accuracy of HbA1c values obtained [5]. One way to investigate this is to focus on glycemic control itself in the acute inpatient setting. As noted by Kristan et al., acute glycemic control may prove to have better outcomes, rendering HbA1c values useless in predicting mortality rates [1]. Interestingly, other studies, including one by Mehta et al., found that there was no correlation between inpatient glycemic control and mortality rates [16]. Taking it a step further, Randhawa et al. found that glycemic control may correlate strongly with severe complications in hospitalized COVID-19 patients but may not affect mortality specifically [18]. Ultimately, our data suggest that chronic outpatient glucose control in the setting of pre-existing diabetes may not correlate with in-hospital mortality in COVID-19 patients admitted to the hospital.

There are numerous avenues for further research into this topic. While particular comorbidities were incorporated into this study, more research could include other comorbidities, including liver and pulmonary diseases, such as cirrhosis and obstructive lung disease. Our study was designed to evaluate a pre-vaccination population and future studies could incorporate data from a broader time period to evaluate a post-vaccination population. Another potential area of focus for future research is the analysis of patients’ outpatient diabetic regimens, including prior insulin or metformin use, and tracking specific inpatient glycemic trends instead of HbA1c values.

A key limitation of our study is its single-centered nature, which solely represents the San Bernardino County population in California. Although similar data may be seen in other regions with a similar population, it is hard to generalize this data throughout all populations and regions. Another limitation of this study was that it was hard to delineate COVID-19 as the primary cause of patients' mortality. It was difficult to rule out other comorbidities and complications during the hospital stay that may have played a significant role in the resulting mortality rates, even though physician discharge/death summaries were meticulously reviewed. That said, it is difficult to say with certainty that every single discharge/death summary reviewed in the study accurately reflected all the comorbidities and chronic conditions that the patient may have had in the outpatient setting. Thus, other unidentified comorbidities could have also played a significant role in mortality rates. Additionally, since the vaccine was excluded as a confounding factor, its presence may have modified the data and could change recommendations about specific individuals’ mortality risks.

## Conclusions

Although comorbidities such as diabetes mellitus can predispose patients to severe COVID-19 disease and even result in hospitalization, there is no clear correlation between poorly controlled diabetes and mortality specifically among patients hospitalized with COVID-19. From this single-center study, higher HbA1c values did not correlate with higher mortality rates among patients who were admitted to the general medical floor or the ICU with a concurrent diagnosis of COVID-19 or respiratory failure due to COVID-19. This suggests that the risk of mortality is not inherently higher in patients with elevated HbA1c values. It also suggests that certain risk factors may not significantly increase the risk of mortality. This type of study can help highlight the need for hospitalization in some patients with COVID-19 regardless of their HbA1c value due to their increased risk of severe infection despite low levels and well-controlled diabetes mellitus. However, further studies are needed to help clinicians delineate whether certain comorbidities are inevitably a greater or lesser risk factor for disease severity in those that acquire COVID-19.
